# Supplementary material for: Room Temperature Incorporation of Arsenic Atoms into the Germanium (001) Surface
Source: Angew Chem Int Ed Engl. 2023 Jan 10;62(7):e202213982. doi: 10.1002/anie.202213982 (PMC10108107; doi:10.1002/anie.202213982)
Supplement: Supplementary file 1 — Supporting Information [file ANIE-62-0-s001.pdf]

Angewandte Chemie
Eine Zeitschrift der Gesellschaft Deutscher Chemiker

Supporting Information

Room Temperature Incorporation of Arsenic Atoms into the Germanium (001) Surface

*E. V. S. Hofmann, T. J. Z. Stock, O. Warschkow, R. Conybeare, N. J. Curson, S. R. Schofield**

Experimental methods

Scanning tunnelling microscopy (STM) experiments were performed in a Scienta Omicron GmbH low-temperature STM system operating at room temperature and under ultrahigh vaccuum $< 5\times10^{-11}$ mbar. High resistivity (1–100 Ωcm antimony doped) germanium 001-oriented samples were degassed overnight at 200°C, then heated to 760°C by direct current heating for 1 hour. The samples were subsequently cleaned by repeated cycles of sputtering (1.2 kV, 10 mA, 30 min) and direct current annealing (700°C, 30 min). Sample preparation was then completed with three 30 s anneals to 760°C, with a 25°C/min cool down from 600°C. An infrared pyrometer (IMPAC IGA50-LO plus) was used to measure the sample temperature, providing absolute temperature measurements accurate to ±30°C. Arsine dosing was performed in-situ with the STM tip macroscopically retracted. The samples were exposed to 0.023 L of 99.999% purity arsine (ATMI Inc.) with a total chamber pressure of 1×10^{-10} mbar. After arsine dosing, the tip was reapproached and imaging started within 15 minutes of dosing.

Density functional theory methodology

Density functional theory (DFT) calculations were performed using the B3LYP hybrid exact-exchange functional,[1,2] atom-centred Gaussian-type orbital basis sets, and methods of energy computations and structure optimisation as implemented in the Gaussian 16 software.[3] A compact $Ge_{21}H_{20}$ cluster model was used to represent the Ge(001) surface, describing three Ge-Ge dimers in the surface-closest atomic layer, and eight, four, and three atoms in the second, third, and fourth atomic layer, respectively. The twenty hydrogen atoms of the cluster provide a chemical termination for all germanium atoms other than those of the surface layer. These cluster-terminating hydrogen atoms were held in fixed positions during all geometry optimisations in order to simulate the strain that would be imposed by the surrounding surface and bulk atoms of an extended surface. All other atoms were fully relaxed during geometry optimisation.

In our calculations two types of composite basis set were used, referred to in the following as *large* and *small*. Geometry optimisations and vibrational frequency calculations were conducted using the small basis, which is composed of the standard 6-311++G(d,p) basis set for all atoms of the adsorbate and the top surface layer of the cluster (i.e. the dimer atoms), a 6-311G(d,p) basis set for second layer atoms, and the core-pseudopotential LANL2DZ basis set for third and fourth layer atoms as well as the cluster-terminating hydrogen atoms. Following geometry optimisation, a single-point energy is calculated at the optimised structure using the large basis set, which is composed of the 6-311++G(2df,2pd) basis set for the adsorbate atoms and top surface layer and a 6-311G(2df,2pd) basis set for all other atoms. Thus, using quantum-chemical notation, the total energy, E, of a given structure is calculated as

E_{SCF}(B3LYP/large//B3LYP/small) + ZPE(B3LYP/small).

Adsorption energies, ΔE, of surface structures arising from the adsorption and dissociation of arsine on Ge(001) were calculated as formation energies of adsorption as follows

$$\Delta E = E_{(\text{cluster+AsH}_3)} - E_{\text{cluster}} - E_{\text{AsH}_3}, \tag{1}$$

where E_{cluster} is the total energy of the bare germanium cluster, $E_{(\text{cluster+AsH}_3)}$ is the total energy of the cluster with an adsorbed or dissociated arsine molecule, and E_{AsH_3} is the total energy of a gas phase arsine molecule.

In some of the adsorption structures considered here, a germanium atom has been displaced away from the adsorption site to a location further away than can be accommodated by our three-dimer cluster. In this case, the adsorption energy is calculated as follows,

$$\Delta E = \frac{2E_{(\text{cluster+AsH}_3-\text{Ge})} + E_{(\text{cluster+2Ge})} - 3E_{\text{cluster}} - 2E_{\text{AsH}_3}}{2}, \tag{2}$$

where $E_{(\text{cluster+AsH}_3-\text{Ge})}$ is the total energy of the cluster with a dissociated arsine molecule and one missing Ge atom and $E_{\text{cluster+2Ge}}$ is the total energy of a cluster with a Ge-Ge ad-dimer, bridging perpendicular between two surface dimers. These ad-dimers are directly observed in our experiments and are therefore the correct reference to account for the displaced germanium atoms in our calculations.

Supporting Information Results

Supporting Information Figure S1a shows a perspective model of the $Ge_{21}H_{20}$ cluster used in our calculations. Details of our computational methodology can be found in the main text. We have calculated a wide range of structures for the adsorption of arsine (AsH_3) on the germanium (001) surface. Supporting Information Figure S1b highlights various adsorption structures; adsorption structures a1, b1, e1, and f1 are the most stable structures we find within the classes of AsH_3, AsH_2 + H, AsH + 2H, and As + 3H, respectively. Supporting Information Figures S1c-f show top view schematics for each of the structures discussed in the main text.

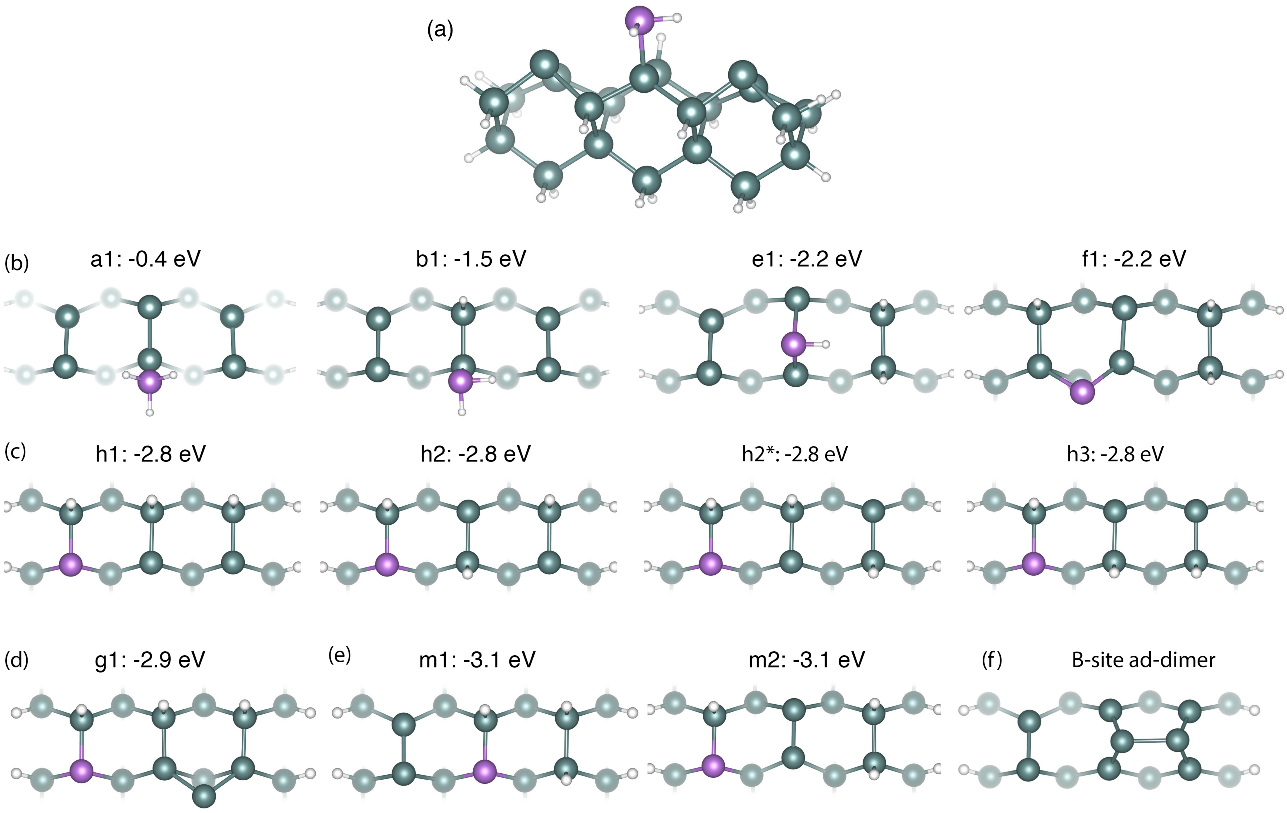

Supporting Information Figure S1: **a,** Perspective view of the $Ge_{21}H_{20}$ cluster used in our calculations. In this illustration an arsine (AsH_3) molecule is shown in an AsH_2 + H dissociative bonding configuration on a single dimer. **b,** Top view models of AsH_3 in various states of dissociation $AsH_x + (3 - x)H$. **c,** Structures h1, h2, h2*, and h3, which all involved surface incorporated arsenic atom forming a hydrogen-terminated As-Ge-H heterodimer and two hemihydride dimers. **d,** Structure g1, equivalent to structure h1 with a captured germanium monomer in an end-bridge configuration. **e,** Structures m1 and m2 that consist of a hydrogen-terminated As-Ge-H heterodimer and a monohydride dimer. **f,** B-site germanium ad-dimer.

References

[1] A. D. Becke, *Phys. Rev. A* **1988**, *38*, 3098–3100.

[2] C. Lee, E. Yang, R. G. Parr, *Phys. Rev. B* **1988**, *37*, 785–789.

[3] M. J. Frisch, G. W. Trucks, H. B. Schlegel, G. E. Scuseria, M. A. Robb, J. R. Cheeseman, G. Scalmani, V. Barone, G. A. Petersson, H. Nakatsuji, X. Li, M. Caricato, A. V. Marenich, J. Bloimo, B. G. Janesko, R. Gomperts, B. Mennucci, H. P. Hratchian, J. V. Ortiz, A. F. Izmaylov, J. L. Sonnenberg, D. Williams-Young, F. Ding, F. Lipparini, F. Egidi, J. Goings, B. Peng, A. Petrone, T. Henderson, D. Ranasinghe, V. G. Zakrzewski, J. Gao, N. Rega, G. Zheng, W. Liang, M. Hada, M. Ehara, K. Toyota, R. Fukuda, J. Hasegawa, M. Ishida, T. Nakajima, Y. Honda, O. Kitao, H. Nakai, T. Vreven, K. Throssell, J. A. Montgomery Jr., J. E. Peralta, F. Ogliaro, M. J. Bearpark, J. J. Heyd, E. N. Brothers, K. N. Kudin, V. N. Staroverov, T. A. Keith, R. Kobayashi, J. Normand, K. Raghavachari, A. P. Rendell, J. C. Burant, S. S. Iyengar, J. Tomasi, M. Cossi, J. M. Millam, M. Klene, C. Adamo, R. Cammi, J. W. Ochterski, R. L. Martin, K. Morokuma, O. Farkas, J. B. Foresman, D. J. Fox, Gaussian, Inc. Wallingford CT, **2016**.
